# Supplementary material for: Musashi–1—A Stemness RBP for Cancer Therapy?
Source: Biology (Basel). 2021 May 5;10(5):407. doi: 10.3390/biology10050407 (PMC8148009; doi:10.3390/biology10050407)
Supplement: Supplementary file 1 [file biology-1199466-supplementary.pdf › biology-1199466-supplementary.pdf]

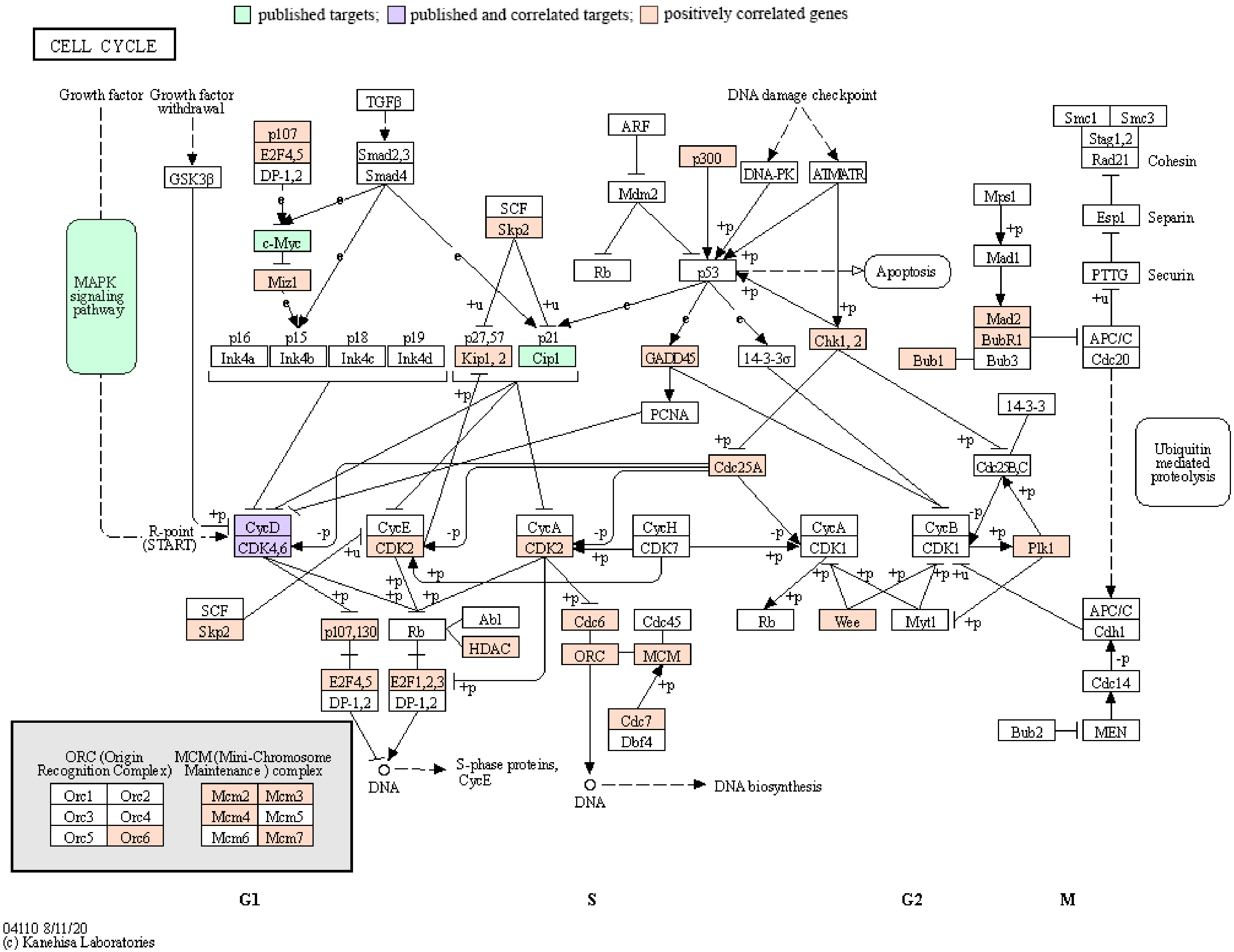

Supplementary Figure S1|**Association of MSI1 expression with KEGG_cell_cycle.** Cell cycle pathway map was created via the Kyoto Encyclopedia of Genes and Genome (KEGG) (https://www.genome.jp/). Published MSI1 target transcripts are depicted in green. Genes that were positively correlated to MSI1 in more than 50% of cancer entities are shown in red. Genes that belong to both categories are shown in purple.
